# Comment on “Efficacy of 7-Day and 14-Day Triple Therapy Regimens for the Eradication of* Helicobacter pylori*: A Comparative Study in a Cohort of Romanian Patients”

**DOI:** 10.1155/2016/1342398

**Published:** 2016-01-21

**Authors:** Amin Talebi Bezmin Abadi

**Affiliations:** Department of Bacteriology, Faculty of Medical Sciences, Tarbiat Modares University, P.O. Box 14115-111, Tehran, Iran

We read with great interest the paper by Arama et al., “Efficacy of 7-Day and 14-Day Triple Therapy Regimens for the Eradication of* Helicobacter pylori*: A Comparative Study in a Cohort of Romanian Patients” [1]. In this randomized prospective study, they compared the eradication rates of* Helicobacter pylori* infection by a 7-day and 14-day anti-HP regimen.* H. pylori* is an infectious disease and the goal of treatment is to cure the infection [2, 3]. This transmissible infection is significantly associated with various digestive diseases and is a main cause of mortality worldwide. Taking together,* H. pylori* is a chronic infectious agent; thus, an ultimate demand is to eradicate it [4]. However, some points we found may help to draw better conclusion.

1. Current reported results are not unexpected within this population. The main limitation of current study is the small sample size which may not support actual representative of this population. Accordingly, a study with larger number of* H. pylori* positive patients is required to draw a better conclusion.
2. The optimal duration for* H. pylori* eradication is still controversial. The authors concluded that two weeks of anti-*H. pylori* regimen is preferable than 7 days. However, they did not mention the rationale for extra side effects due to the increased duration of this therapy. So, it can be a good opportunity to rethink about optimal* H. pylori* treatment duration.
3. Being cost-effective is an important factor for an optimum duration of* H. pylori* eradication regimens. However, this item is influencing 14-day treatment.

Conclusively, an optimal first-line* H. pylori* eradication therapy has to be discovered already. Certainly, preantibiotic susceptibility tests are inevitable approach in* H. pylori* therapy.
